# Supplementary figures and images for: Structural and biophysical properties of FopA, a major outer membrane protein of Francisella tularensis
Source: PLoS One. 2022 Aug 1;17(8):e0267370. doi: 10.1371/journal.pone.0267370 (PMC9342783; doi:10.1371/journal.pone.0267370)

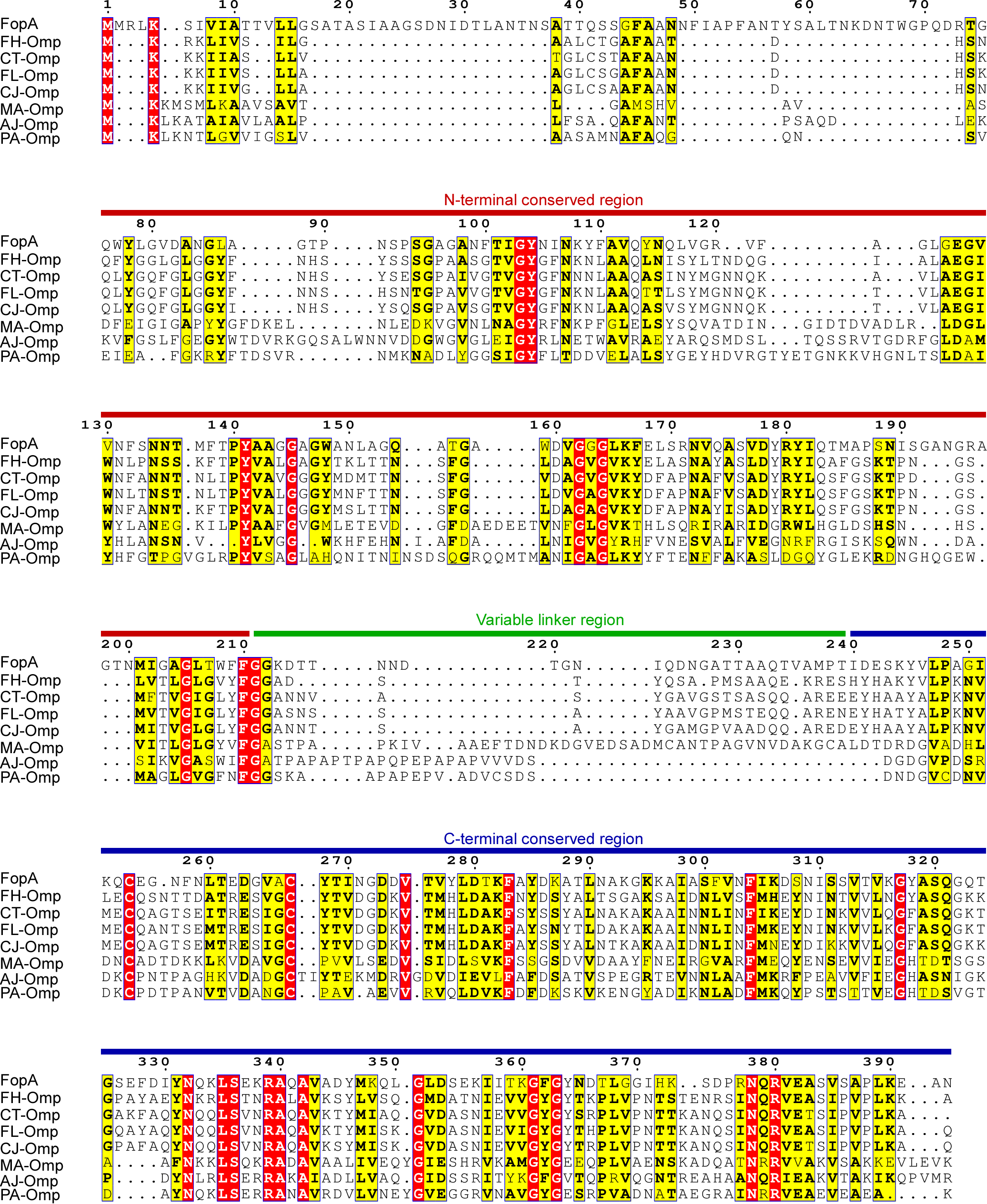

Supplement: S1 Fig — From top to bottom, the proteins are from Fangia hongkongensis (FH), Caedibacter taeniospiralis (CT), Fastidiosibacter lacustris (FL), Cysteiniphilum sp. JM-1 (CJ), Marinagarivorans algicola (MA), Alishewanella jeotgali (AJ) and Pseudomonas aeruginosa (PA). The sequence numbering is for FopA. Residues that are 100% conserved are in red background with white text. For a given position, if 70% of the amino acids are identical or similar, these residues are highlighted in yellow, with the similar residues in bold. Top horizontal lines indicate two conserved regions localized to the N- and C-terminals (red and blue lines respectively) that are dispersed by a highly variable region (green line). (TIF) [file pone.0267370.s001.tif]

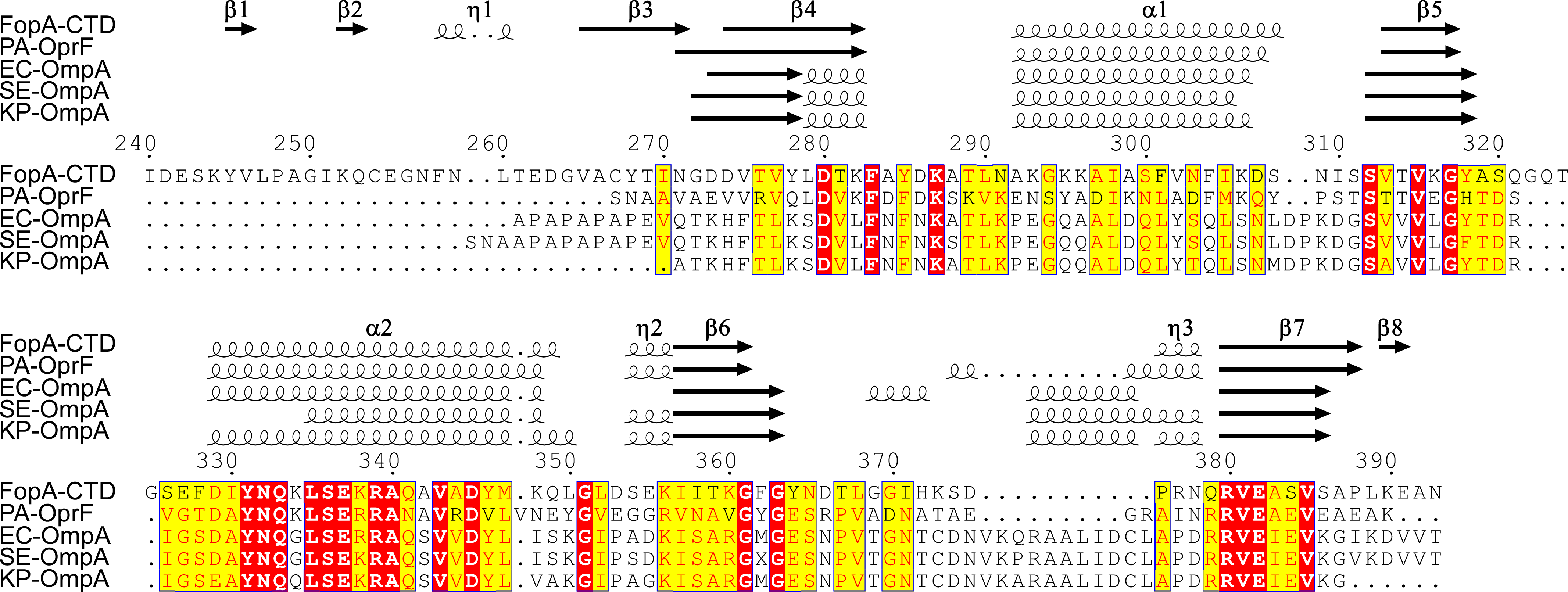

Supplement: S2 Fig — The secondary structural elements of each OMP-CTD are shown above the sequences. The sequence numbering is for FopA. The OMPs used in the analysis are: Pseudomonas aeruginosa (PA) OprF-CTD (PDB ID 5U1H), Escherichia coli (EC) OmpA-CTD (PDB ID 2MQE), Salmonella enterica (SE) OmpA-CTD (PDB ID 4ERH) and Klebsiella pneumoniae (KP) OmpA-CTD (PDB ID 5NHX). Residues that are 100% conserved are in red background with white text. For a given position, if 70% of the amino acids are identical or have similar physico-chemical properties, they are highlighted in yellow, with similar residues in red characters. β-strands (black arrows), α-helices (spirals) and 310-helices (η) are indicated. Gaps are represented by black dots. (TIF) [file pone.0267370.s002.tif]

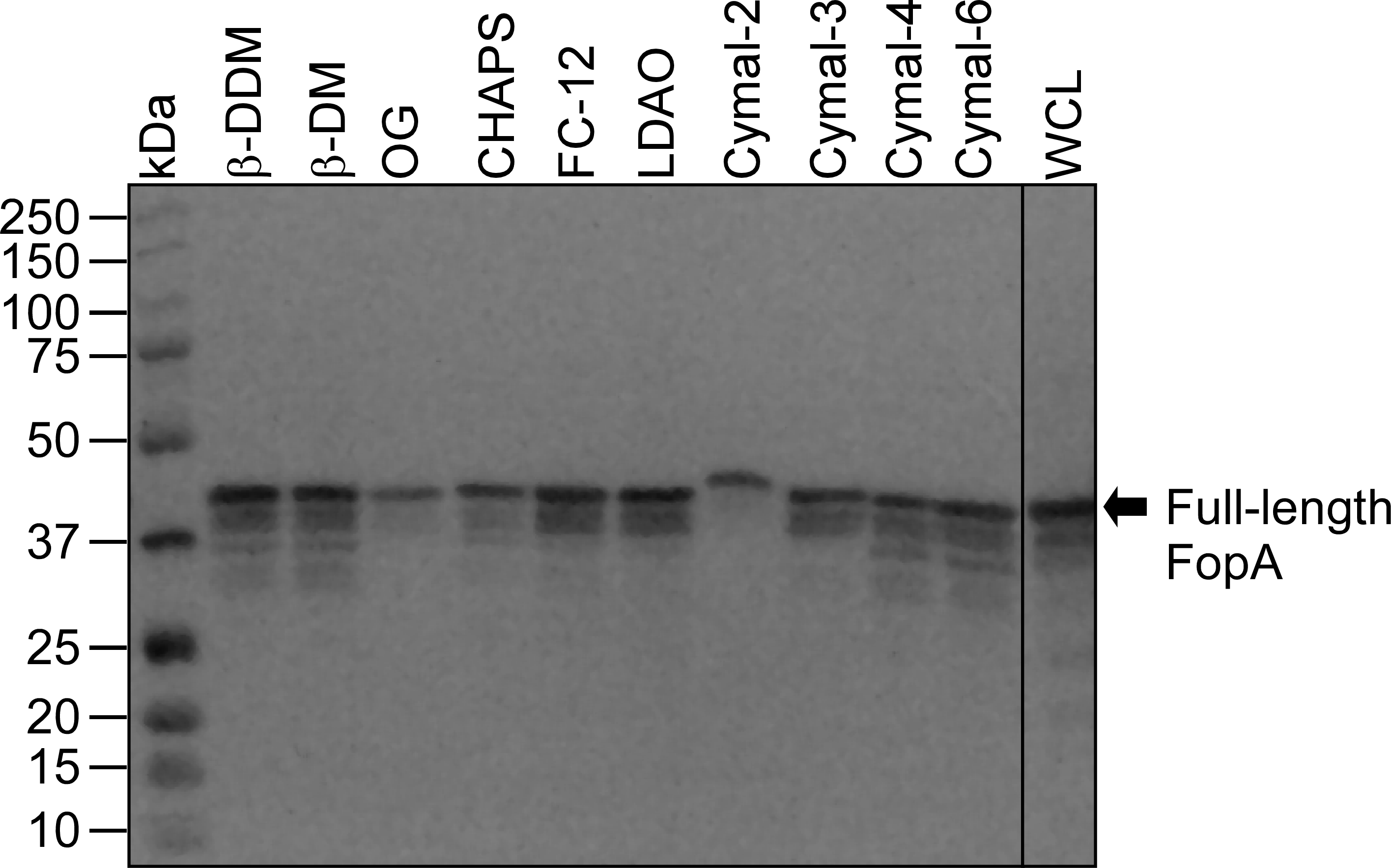

Supplement: S3 Fig — Each lane in this anti-FopA immunoblot was loaded with the detergent-solubilized supernatant from an E. coli membrane fraction, except for the leftmost and rightmost lanes, which were loaded with MW size standards and whole cell lysate (WCL), respectively. No detergent was added to the WCL. Full-length FopA is indicated by a black arrow. (TIF) [file pone.0267370.s003.tif]

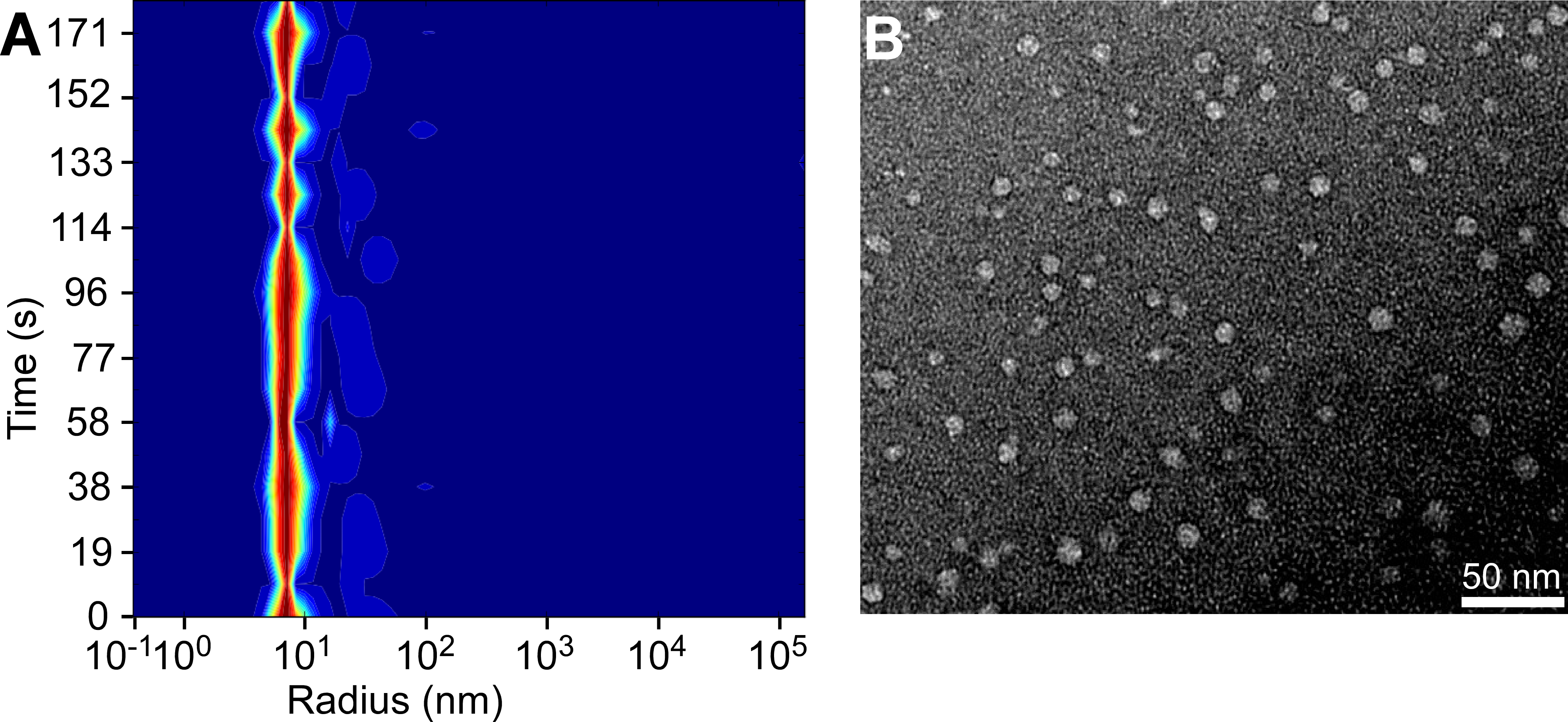

Supplement: S4 Fig — (A) Dynamic light scattering and (B) negative stain electron microscopy. Both analysis reveals a homogenous distribution of particles with a size range of 8–10 nm. (TIF) [file pone.0267370.s004.tif]

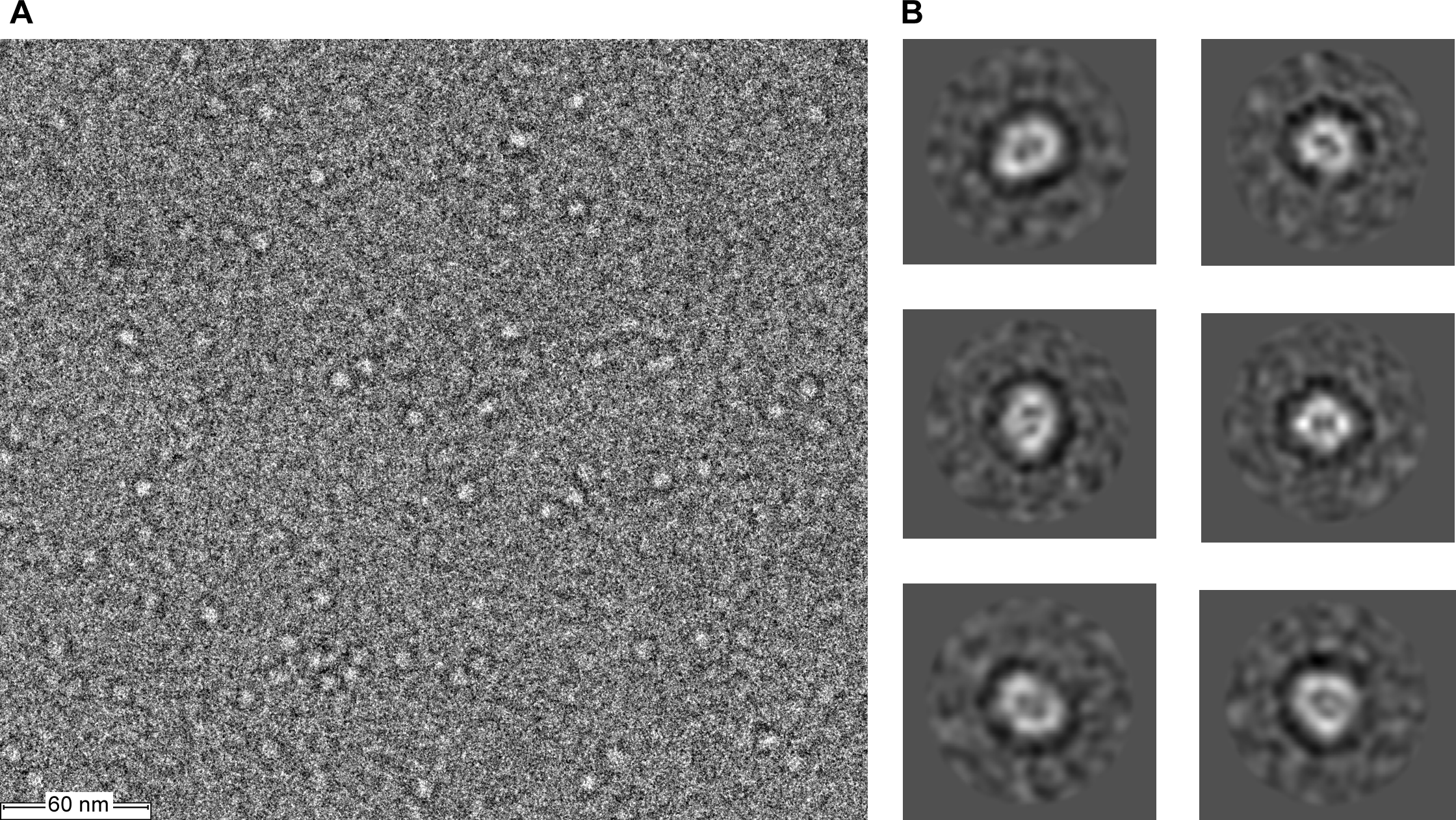

Supplement: S5 Fig — (A) An EM micrograph of negatively stained FopA particles. (B) Representative 2D class averages. (TIF) [file pone.0267370.s005.tif]

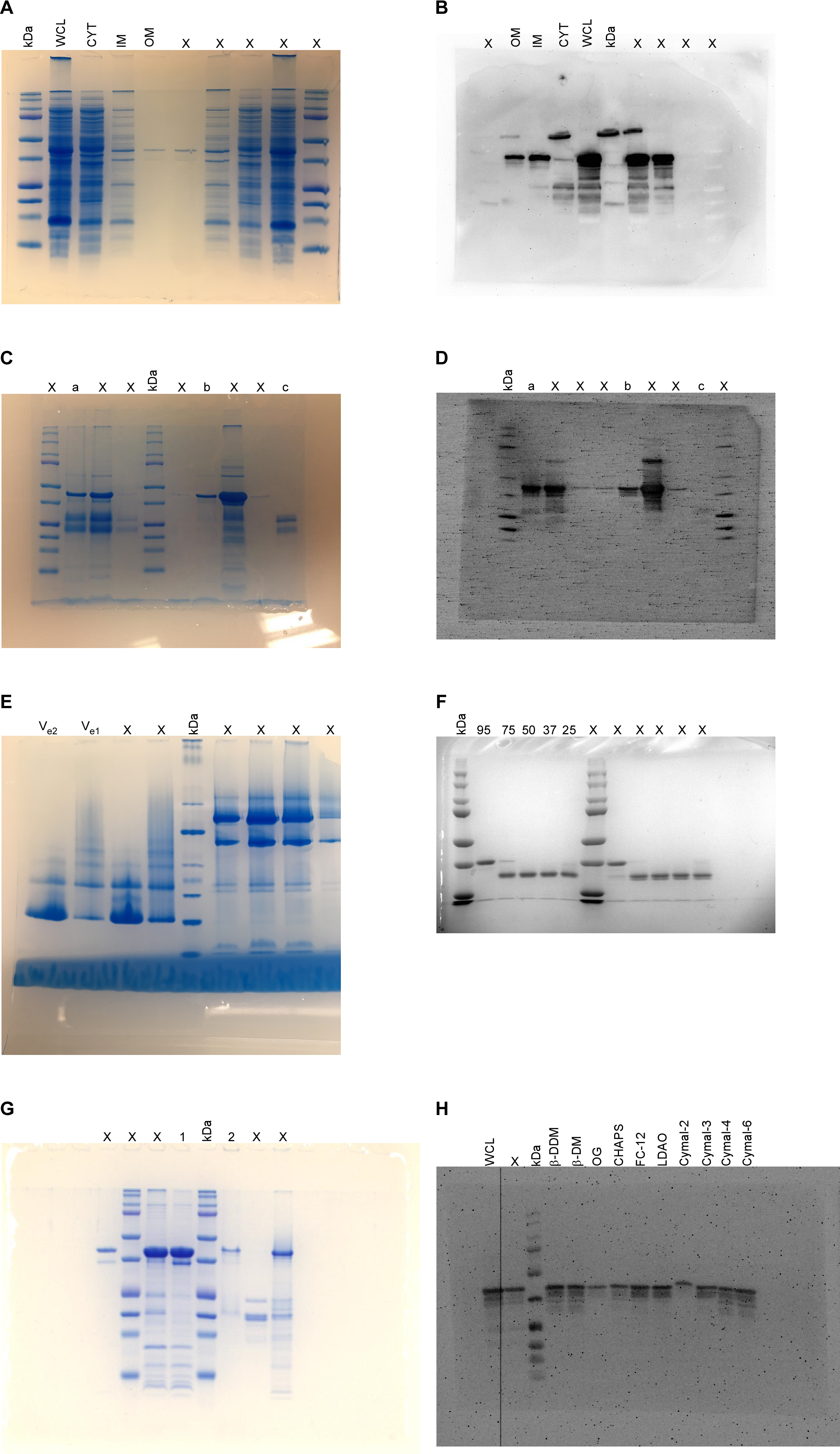

Supplement: S1 Raw image — (A) Uncropped gel image corresponding to Fig 2B captured by cell phone camera. (B) Uncropped blot image corresponding to Fig 2C captured by Kodak Gel Logic 440 gel imaging system. (C) Uncropped gel image corresponding to Fig 3C captured by cell phone camera. (D) Uncropped blot image corresponding to Fig 3D captured by Bio-Spectrum Imaging System (UVP, LLC, Upland, CA). (E) Uncropped gel image corresponding to Fig 4B captured by cell phone camera. (F) Uncropped gel image corresponding to Fig 5B captured by UVP–Gel studio plus gel doc system. (G) Uncropped gel image corresponding to Fig 8B captured by Bio-Spectrum Imaging System (UVP, LLC, Upland, CA). (H) Uncropped blot image corresponding to S3 Fig captured by Bio-Spectrum Imaging System (UVP, LLC, Upland, CA). (TIF) [file pone.0267370.s010.tif]
