# Supplementary material for: Structural and biophysical properties of FopA, a major outer membrane protein of Francisella tularensis
Source: PLoS One. 2022 Aug 1;17(8):e0267370. doi: 10.1371/journal.pone.0267370 (PMC9342783; doi:10.1371/journal.pone.0267370)
Supplement: S1 Table — a Listed are all of the homologs with at least 25% sequence identity to FopA. b Percentage of query sequence residues that overlap with the reference sequence. c Expect value (E) is a parameter that describes the number of hits one can "expect" to see by chance when searching a database. d Quantitative measurement of the similarity between two sequences. (PDF) [file pone.0267370.s006.pdf]

Table S1. Closest FopA homologs identified by BLAST search [a].

Organism	NCBI Reference Sequence	Order	Family	Query cover[b]	E value[c]	Identity [d]
Fangia hongkongensis	WP_018300465.1	Thiotrichales	Fastidiosibacteraceae	76%	4e-55	39.22%
Caedibacter taeniospiralis	WP_100550987.1	Thiotrichales	Fastidiosibacteraceae	82%	5e-52	36.28%
Fastidiosibacter lacustris	WP_116964154.1	Thiotrichales	Fastidiosibacteraceae	82%	6e-52	35.98%
Cysteiniphilum sp. JM-1	WP_151194100.1	Thiotrichales	Fastidiosibacteraceae	81%	6e-52	36.89%
Geobacter bemidjiensis	WP_012529806.1	Desulfuromonadales	Geobacteraceae	75%	2e-13	25.94%
Marinagarivorans algicola	WP_053981259.1	Cellvibrionales	Cellvibrionaceae	75%	3e-12	26.28%
Alishewanella jeotgali	WP_008951022.1	Alteromonadales	Alteromonadaceae	86%	7e-12	26.46%
Pseudomonas aeruginosa	WP_134304455.1	Pseudomonadales	Pseudomonadaceae	64%	7e-11	28.09%
Marinomonas piezotolerans	WP_115468919.1	Oceanospirillales	Oceanospirillaceae	67%	1e-09	26.41%

[a] Listed are all of the homologs with at least 25% sequence identity to FopA.
[b] Percentage of query sequence residues that overlap with the reference sequence.
[c] Expect value (E) is a parameter that describes the number of hits one can "expect" to see by chance when searching a database.
[d] Quantitative measurement of the similarity between two sequences.
