# Supplementary material for: Structural and biophysical properties of FopA, a major outer membrane protein of Francisella tularensis
Source: PLoS One. 2022 Aug 1;17(8):e0267370. doi: 10.1371/journal.pone.0267370 (PMC9342783; doi:10.1371/journal.pone.0267370)
Supplement: S2 Table — (PDF) [file pone.0267370.s007.pdf]

Table S2. FopA secondary structure estimated by bioinformatics and CD spectroscopy.

Analysis method	Helices (%)	Strands (%)	Turns (%)	Unordered (%)
Phyre^2 prediction	22	32	-	30
CD spectroscopy	17.3	30.6	21.6	29.7
