# Supplementary material for: Structural and biophysical properties of FopA, a major outer membrane protein of Francisella tularensis
Source: PLoS One. 2022 Aug 1;17(8):e0267370. doi: 10.1371/journal.pone.0267370 (PMC9342783; doi:10.1371/journal.pone.0267370)
Supplement: S3 Table — (PDF) [file pone.0267370.s008.pdf]

Table S3. **FopA crystals X-ray diffraction.**

Data collection and processing statistics	
Beamline	23-ID-D
Crystal size (μm)	25 x 25 x 1
Temperature (K)	100
Wavelength (Å)	1.033
Maximum Resolution observed (Å)	5
Resolution range (Å)	42.0-6.5
Space group	H32
Unit cell dimensions	
a, b, c (Å)	48.9, 48.9, 324.5
α, β, γ (°)	90, 90, 120
Completeness (%)	98.1
Overall I/σ	9.04
Total number of reflections	10,651
Number of unique reflections	663
