# Supplementary material for: Structural and biophysical properties of FopA, a major outer membrane protein of Francisella tularensis
Source: PLoS One. 2022 Aug 1;17(8):e0267370. doi: 10.1371/journal.pone.0267370 (PMC9342783; doi:10.1371/journal.pone.0267370)
Supplement: S1 File — (A) Protein sequence. (B) Plasmid DNA sequence. (PDF) [file pone.0267370.s009.pdf]

S1 File. Expression clone detail. (A) Protein sequence. (B) Plasmid DNA sequence.

A

LOCUS Translation\of\pRSET-FTT0583-His8 401 aa 25-OCT-2018
SOURCE
ORGANISM
FEATURES Location/Qualifiers
Site 394..401
/vntifkey="264"
/label=**His8**
Region 1..393
/vntifkey="1000"
/label=**FopA\full-length**
Region 1..23
/vntifkey="200"
/label=**signal\sequence**
ORIGIN
1 **mmrlksivia ttvllgsata siaagsdnid tlantnsatt qssgfaannf iapfantysa**
61 **ltnkdntwgp qdrtgqwylg vdanglagtp nspsgaganf tigyninkyf avqynqlvgr**
121 **vfaglgegvv nfsnntmftp yaaggagwan lagqatgawd vggglkfels rnvqasvdyr**
181 **yiqtmapsni sgangragtn migagltwff ggkdttnndt gniqdngatt aaqtvampti**
241 **deskyvlpag ikqcegnfnl tedgvacyti ngddvtvyld tkfaydkatl nakgkkaias**
301 **fvnfikdsni ssvtvkgyas qgqtgsefdi ynqklsekra qavadymkql gldsekiitk**
361 **gfgyndtlgg ihksdprnqr veasvsaplk eanhhhhhhh h**
//

B

LOCUS pRSET-FTT0583-His8 3995 bp DNA circular 9-OCT-2018
SOURCE
ORGANISM
FEATURES Location/Qualifiers
promoter 20..39
/vntifkey="30"
/label=**T7\promoter**
rep_origin 1554..2009
/vntifkey="33"
/label=f1\ori
CDS 2140..3000
/vntifkey="4"
/label=AMP-R
promoter 2041..2139
/vntifkey="30"
/label=bla\pr
rep_origin 3145..3818
/vntifkey="33"
/label=pUC\ori
RBS 85..92
/vntifkey="32"
/label=RBS
terminator 1354..1483
/vntifkey="43"
/label=T7\term
CDS 1279..1302
/vntifkey="4"
/label=**His8**
CDS 100..1278
/vntifkey="4"
/label=**FopA**
BASE COUNT 1069 a 890 c 949 g 1087 t
ORIGIN
1 gatctcgatc ccgcgaaat**t aatacgactc actata**ggga gaccacaacg gtttccctct
61 agaaataatt ttgtttaact ttaagaagga gatatacat**t tgatgagatt aaaaagtatt**
121 **gttatagcta caactgtatt attaggttca gctacagcat ctatcgctgc aggttcagat**
181 **aatatcgata cattagcaaa cactaattca gctactacac aaagcagtgg ttttgcagct**
241 **aataatttca ttgctccttt tgcaaatact tatagcgctt tgactaacaa ggacaatact**
301 **tggggtcctc aagatagaac tggccagtgg tacttaggtg tagatgctaa cggtctagct**
361 **ggaactccta actctccatc aggtgctggt gctaacttca caatcggtta taacatcaat**
421 **aaatacttcg ctgtacagta caaccaatta gttggtagag tatttgctgg tttaggtgaa**
481 **ggtgttgtaa actttagtaa taatactatg tttactccat atgctgcagg tggtgctggt**
541 **tgggcaaatc tagcaggtca agcaacaggt gcttgggatg tgggtggtgg tcttaagttt**
601 **gaactatcta gaaatgttca agcaagtgtt gactacagat atatccaaac aatggcacct**
661 **agtaatattt ctggtgctaa tggcagagcg ggtactaaca tgattggtgc tggtttaaca**
721 **tggttctttg gtggcaaaga tactactaat aatgacactg gtaatattca ggataatggt**
781 **gcgactacag ctgctcaaac tgttgctatg ccaactattg atgagtctaa gtatgtttta**
841 **cctgctggta ttaagcaatg tgaaggcaac tttaatctaa ctgaagatgg tgtcgcgtgc**
901 **tatacaataa atggtgatga tgtaacagtt tacctagata ctaagtttgc ttatgataaa**
961 **gctactttaa atgctaaagg taaaaaagct attgcatctt ttgttaattt tatcaaggat**
1021 **agtaacatta gctctgtaac agttaaaggt tatgcttctc aaggtcaaac tggtagcgag**

1081 **tttgatatat ataaccaaaa actttctgag aagagagcac aagctgttgc tgattacatg**
1141 **aagcaattag gtttagatag tgagaaaata attactaaag gttttggcta taatgatact**
1201 **ttaggtggta ttcataagtc tgatccgcgt aaccagcgtg tagaagctag cgtatcagct**
1261 **ccacttaaag aagctaacca ccatcatcac caccatcacc at**taatagaa gggcgaattc
1321 cagcacactg gcggccgtta ctagtggatc cggctgctaa caaagcccga aaggaagctg
1381 agttggctgc tgccaccgct gagcaataac tagcataacc ccttggggcc tctaaacggg
1441 tcttgagggg ttttttgctg aaaggaggaa ctatatccgg atctggcgta atagcgaaga
1501 ggcccgcacc gatcgccctt cccaacagtt gcgcagcctg aatggcgaat gggacgcgcc
1561 ctgtagcggc gcattaagcg cggcgggtgt ggtggttacg cgcagcgtga ccgctacact
1621 tgccagcgcc ctagcgcccg ctcctttcgc tttcttccct tcctttctcg ccacgttcgc
1681 cggctttccc cgtcaagctc taaatcgggg gctcccttta gggttccgat ttagtgcttt
1741 acggcacctc gaccccaaaa aacttgatta gggtgatggt tcacgtagtg ggccatcgcc
1801 ctgatagacg gtttttcgcc ctttgacgtt ggagtccacg ttctttaata gtggactctt
1861 gttccaaact ggaacaacac tcaaccctat ctcggtctat tcttttgatt tataagggat
1921 tttgccgatt tcggcctatt ggttaaaaaa tgagctgatt taacaaaaat ttaacgcgaa
1981 ttttaacaaa atattaacgc ttacaattta ggtggcactt ttcggggaaa tgtgcgcgga
2041 acccctattt gtttattttt ctaaatacat tcaaatatgt atccgctcat gagacaataa
2101 ccctgataaa tgcttcaata atattgaaaa aggaagagta tgagtattca acatttccgt
2161 gtcgccctta ttcccttttt tgcggcattt tgccttcctg tttttgctca cccagaaacg
2221 ctggtgaaag taaaagatgc tgaagatcag ttgggtgcac gagtgggtta catcgaactg
2281 gatctcaaca gcggtaagat ccttgagagt tttcgccccg aagaacgttt tccaatgatg
2341 agcactttta aagttctgct atgtggcgcg gtattatccc gtattgacgc cgggcaagag
2401 caactcggtc gccgcataca ctattctcag aatgacttgg ttgagtactc accagtcaca
2461 gaaaagcatc ttacggatgg catgacagta agagaattat gcagtgctgc cataaccatg
2521 agtgataaca ctgcggccaa cttacttctg acaacgatcg gaggaccgaa ggagctaacc
2581 gcttttttgc acaacatggg ggatcatgta actcgccttg atcgttggga accggagctg
2641 aatgaagcca taccaaacga cgagcgtgac accacgatgc ctgtagcaat ggcaacaacg
2701 ttgcgcaaac tattaactgg cgaactactt actctagctt cccggcaaca attaatagac
2761 tggatggagg cggataaagt tgcaggacca cttctgcgct cggcccttcc ggctggctgg
2821 tttattgctg ataaatctgg agccggtgag cgtgggtctc gcggtatcat tgcagcactg
2881 gggccagatg gtaagccctc ccgtatcgta gttatctaca cgacggggag tcaggcaact
2941 atggatgaac gaaatagaca gatcgctgag ataggtgcct cactgattaa gcattggtaa
3001 ctgtcagacc aagtttactc atatatactt tagattgatt taaaacttca tttttaattt
3061 aaaaggatct aggtgaagat cctttttgat aatctcatga ccaaaatccc ttaacgtgag
3121 ttttcgttcc actgagcgtc agaccccgta gaaaagatca aaggatcttc ttgagatcct
3181 ttttttctgc gcgtaatctg ctgcttgcaa acaaaaaaac caccgctacc agcggtggtt
3241 tgtttgccgg atcaagagct accaactctt tttccgaagg taactggctt cagcagagcg
3301 cagataccaa atactgttct tctagtgtag ccgtagttag gccaccactt caagaactct
3361 gtagcaccgc ctacatacct cgctctgcta atcctgttac cagtggctgc tgccagtggc
3421 gataagtcgt gtcttaccgg gttggactca agacgatagt taccggataa ggcgcagcgg
3481 tcgggctgaa cggggggttc gtgcacacag cccagcttgg agcgaacgac ctacaccgaa
3541 ctgagatacc tacagcgtga gctatgagaa agcgccacgc ttcccgaagg gagaaaggcg
3601 gacaggtatc cggtaagcgg cagggtcgga acaggagagc gcacgaggga gcttccaggg
3661 ggaaacgcct ggtatcttta tagtcctgtc gggtttcgcc acctctgact tgagcgtcga
3721 tttttgtgat gctcgtcagg ggggcggagc ctatggaaaa acgccagcaa cgcggccttt
3781 ttacggttcc tggccttttg ctggcctttt gctcacatgt tctttcctgc gttatcccct
3841 gattctgtgg ataaccgtat taccgccttt gagtgagctg ataccgctcg ccgcagccga
3901 acgaccgagc gcagcgagtc agtgagcgag gaagcggaag agcgcccaat acgcaaaccg
3961 cctctccccg cgcgttggcc gattcattaa tgcag
//
